# Memory span measured by the spatial span tests of the Cambridge Neuropsychological Test Automated Battery in a group of Brazilian children and adolescents

**DOI:** 10.1590/S1980-57642011DN05020012

**Published:** 2011

**Authors:** Rosani Aparecida Antunes Teixeira, Elaine Cristina Zachi, Daniela Tsubota Roque, Anita Taub, Dora Fix Ventura

**Affiliations:** 1 Instituto de Psicologia, Universidade de São Paulo, São Paulo SP, Brazil; 2 Instituto de Psiquiatria, Faculdade de Medicina da Universidade de São Paulo, São Paulo SP, Brazil

**Keywords:** neuropsychological tests, CANTAB, spatial span, working memory, children, educational status

## Abstract

The neuropsychological tests of spatial span are designed to measure attention and working memory. The version of the spatial span test in the Cambridge Neuropsychological Test Automated Battery (CANTAB) evaluates these functions through the recall of sequences of spatial locations presented to the subject.

**Objective:**

The present study investigated how age, gender and educational level might affect the performance of the non-verbal system.

**Methods:**

A total of 60 children and adolescents aged 6 to 18 years were assessed (25 males and 35 females).

**Results:**

The results showed no gender differences in test performance. Children with six or more years of education showed better performance than children with less than three years of education. Older children had more schooling and thus were able to recall a greater number of items. Span length values proved similar to a previous large normative study which also employed the CANTAB Spatial Span (De Luca et al., 2003).

**Conclusion:**

The similarity in performance of the Brazilian children and adolescents studied and the group of Australian participants examined by the cited authors, despite the socio-cultural and economical differences, points to the suitability of the task for the assessment of attention and working memory in Brazilian children.

## Abstract (pt)

Testes neuropsicológicos de span visuoespacial são construídos para avaliar amplitude atencional e memória de trabalho. Na versão do teste de span visuoespacial da Cambridge Neuropsychological Test Automated Battery (CANTAB) essa avaliação é feita através de sequências de localização espacial.

**Objetivo:**

O presente estudo investiga como a idade, o sexo e a escolaridade podem afetar o desempenho nesse sistema não-verbal.

**Métodos:**

Foram avaliadas 60 crianças e adolescentes com idades entre 6 e 18 anos (25 meninos; 35 meninas).

**Resultados:**

Os resultados não mostraram diferenças no desempenho do teste entre os sexos. O grupo de crianças com seis ou mais anos de escolaridade foi melhor do que a de crianças menores de três anos de estudo. Nossos resultados sugerem que as crianças mais velhas e, portanto, com maior tempo de escolaridade conseguem se lembrar de um número maior de itens. Os valores de amplitude foram similares aos obtidos em um experimento normativo prévio amplo no qual foi utilizado o teste Spatial Span do CANTAB (De Luca et al., 2003).

**Conclusão:**

Estas concordâncias de desempenhos entre crianças e adolescentes brasileiros e o grupo de participantes australianos examinados pelos autores citados nos mostram que apesar das diferenças sócio-culturais e econômicas existentes, a tarefa é adequada para avaliação de atenção e memória de trabalho em crianças brasileiras.

Memory span is “the ability to grasp a number of dizscrete units in a single moment of attention and to reproduce them immediately”.^1^ Memory span assessment measures the ability to recall series of discrete stimuli, such as digits, letters, words, sounds, immediately after their presentation. Practically every type of material can be used in test span capacity.^1^

The Spatial Span Test is frequently considered a nonverbal analogue of the Digit Span Test, which measures the capacity of visuospatial memory.^2^ The most frequently cited theoretical model in recent research on verbal short-term memory development has been the Baddeley and Hitch^3^ working memory model.

Working memory is a limited capacity system serving to keep “active” a limited amount of information for a brief period of time, and then to operate on it. The Baddeley and Hitch working memory model includes a central component, the central executive, and three sub-systems: the phonological loop, the visuospatial sketchpad and the episodic buffer.^4^

In its original formulation^3^ this model proposed the existence of two separate systems involved in working memory. These two systems handled different classes of information: the articulatory loop handles speech-based (phonological) information that allows auditory information to be held through a rehearsal mechanism that prevents its rapid decay, and the visuospatial sketchpad handles visual (e.g., color) and spatial (e.g., location) information allowing this information to be maintained and manipulated.^4^

The episodic buffer is an addition to the original working memory model. It was proposed by Baddeley in order to handle phenomena that were not covered by the first model. In the revised model he intended to bring together and integrate the information from the other components of working memory, together with information about time and order.^4^

The present study is concerned with memory for spatial information and therefore assesses the visuospatial sketchpad proposed by Baddeley and Hitch. The aim was to investigate how age, gender and educational level might affect the performance of the non-verbal system, using a modern computerized instrument called the Cambridge Neuropsychological Test Automated Battery (CANTAB) (Cambridge Cognition, 2005). The CANTAB was developed over 20 years ago at the University of Cambridge by Robbins and Sahakian^5^ for evalutation of cognitive function.

The CANTAB is a computerized neuropsychological battery consisting of 22 tests for assessing memory, attention and executive function. This software tool has been widely used since the 1990s. Subjects are tested using a touch-screen computerized system that provides an accurate neuropsychological assessment and latency recording. Some tests require a press pad, mainly those measuring reaction time.

The Spatial Span test is a computerized version of the Corsi Block Tapping Task^6^ and assesses the ability to remember a sequence of squares lighting up on the screen. The Corsi Block Tapping task was developed in the early 1970s as a visuospatial counterpart to the verbal-memory span task and has frequently been used to assess visuospatial short-term memory performance in adults, children and patients with neuropsychological deficits. Vandierendonck and collaborators^7^ explored the information-processing operations measured by the Corsi Blocks Tapping task within the working-memory framework developed by Baddeley and Hitch.^3^

## Methods

Subjects included 60 children and adolescents aged 6 to 18 years (9.26±2.79ys) with varying levels of formal education (3.91±2.55 years). The children were recruited from the Escola de Aplicação, Universidade de São Paulo, São Paulo, Brazil. Exclusion criteria were history of head injury and/or psychiatric illnesses. The procedures were approved by the Human Research Ethics Committee of the University Hospital of the University of São Paulo (SISNEP CAAE: 0026.0.198.000-06) and written informed consent was obtained from participants or their guardians, prior to testing.

The demographic characteristics of the participants are summarized in Table 1.

**Table 1:** Demographic features of children and adolescents included in the study.

AGE (yrs)	Total N	Males	Females	Years of schooling (SD)
6	7	4	3	1 (0)
7	10	4	6	1.8 (0.42)
8	8	2	6	2.57 (0.53)
9	8	3	5	3.25 (0.46)
10	13	3	10	4.35 (0.49)
11	5	3	2	5 (0)
12-18	9	5	4	8.22 (2.33)
Total	60	24	36	

SD: standard deviation.

The Spatial Span Test is a test from the Cambridge Neuropsychological Test Automated Battery (CANTABeclipse)^8^ that assesses working memory capacity, and is a visuospatial analogue of the Digit Span test. In the Spatial Span Test, white squares are shown, some of which briefly change colour in a variable sequence. For each trial, nine randomly arranged white squares are shown on the screen. One by one the squares light up in colour, in a variable sequence and children were instructed to remember the sequence. At the end of the presentation, the children are required to touch each of the boxes that had lit up, in the same order in the first part of the test, and again in reverse order in the second part. The task begins with the simplest level of a two box sequence. After each successful trial, the number of boxes in the sequence is increased one by one to a maximum of nine. If the child’s response was incorrect at any particular level, an alternate sequence of the same length was presented. The test is terminated when the child fails three consecutive trials at any one level.^9^

The measure obtained was the longest visual span, defined by the maximum number of boxes the subject correctly touched.

## Data analysis

To test whether the data were normally distributed, Kolmogorov-Smirnov tests were run (Z=0.65, not significant). Intergroup comparison between gender groups and among different schooling levels were performed by analysis of variance (ANOVA). Associations between span scores and age were analyzed using regression models. The significance level used was 0.05.

## Results

No statistical differences were found between male (n=24) and female (n=36) participants’ results on the visual span test for the items span forward and backward, attempts forward and backward, or errors forward and backward (Table 2).

**Table 2:** Performance by gender.

Sex (n)	Span forward	Span backward	Attempts forward	Attempts backward	Errors forward	Errors backward
Male (24)	5.04 (1.54)	4.92 (1.65)	7.92 (2.30)	7.96 (2.35)	12.20 (6.01)	12.12 (5.39)
Female (36)	5.02 (1.36)	4.70 (1.65)	7.6 (1.81)	7.37 (2.26)	12.22 (5.02)	10.94 (5.01)

Mean (standard deviation).

Children and adolescents were divided into groups according to years of schooling. Group 1 was comprised by children with only one year of schooling, Group 2 by those with two years of schooling, Group 3 three years of schooling, Group 4 four years of schooling, Group 5 five years of schooling, and Group 6 by children with six or more years of schooling.

When the results of the same test were compared, no statistical difference between the scores of forward and backward Spatial Span was observed (p≥0.05) (Table 3).

**Table 3:** Performance by years of schooling.

Years of schooling(n)	Forward Span Mean (SD)	Backward Span Mean (SD)	P-Value	Forward attempts Mean (SD)	Backward attempts Mean (SD)	Forward errors Mean (SD)	Backward errors Mean (SD)
1 (9)	3.66 (0.5)	3.33 (0.86)	≥0.05	6.0 (1.0)	5.66 (1.32)	10.33 (3.27)	8.33 (2.91)
2 (11)	4.63 (1.2)	4.45 (1.36)	≥0.05	7.0 (1.48)	7.18 (1.94)	11.63 (5.42)	10.91 (4.64)
3 (10)	4.8 (1.13)	4.3 (1.41)	≥0.05	7.40 (1.07)	7.70 (3.23)	11.80 (2.89)	12.40 (7.64)
4 (11)	5.2 (1.13)	4.8 (0.78)	≥0.05	8.70 (2.05)	7.50 (1.35)	14.90 (7.26)	12.0 (4.89)
5 (9)	5.2 (1.39)	5.1 (1.10)	≥0.05	8.0 (2.21)	7.70 (1.70)	12.10 (4.38)	10.60 (3.47)
≥6 (10)	6.6 (1.57)	6.3 (1.76)	≥0.05	9.40 (2.27)	9.80 (2.09)	12.40 (7.54)	14.20 (5.30)

SD (standard deviation).

The numbers of attempts increased with age, while the numbers of errors decreased with age in almost all groups (Table 4).

**Table 4:** Performance of different age groups.

Age (n)	Forward attemptsMean (SD)	Backward attemptsMean (SD)	Forward errorsMean (SD)	Backward errorsMean (SD)
6 (7)	3.57 (0.53)	3.28 (0.95)	9.71 (2.36)	9.00 (2.94)
7 (10)	4.50 (1.80)	4.50 (1.35)	12.20 (6.01)	10.60 (4.99)
8 (8)	5.00 (0.75)	4.00 (0.92)	13.12 (4.76)	10.50 (3.96)
9 (8)	4.87 (1.45)	4.50 (1.69)	12.12 (4.29)	11.87 (8.23)
10 (13)	5.30 (0.94)	4.84 (0.55)	14.46 (6.09)	11.61 (4.50)
11 (5)	4.80 (1.78)	5.40 (1.34)	9.61 (2.07)	11.20 (4.54)
≥12 (9)	6.66 (1.65)	6.44 (1.81)	11.66 (7.61)	14.55 (5.50)

SD (standard deviation).

With regard to the variable years of schooling, statistical differences were found between groups with 1 yr (p<0.01), 2 yrs (p<0.01), 3 yrs (p=0.01) versus 6 yrs on forward span scores, and also between the 1 yr (p<0.01), 2 yrs (p=0.01), 3 yrs (p=0.01), 4 yrs (p=0.02) groups versus the 6 year Group, on the backward span scores.

The analysis as a function of age compared the following groups: 6 year-old children, 7-year-olds, 8-year-olds, 9-year-olds, 10-year-olds, 11-year-olds and 12-18 year-old children.

Statistical differences were found for age between the group of 6-year-olds (p=0.04) compared with 10 and 12-18 year-olds, 7 year-olds (p<0.01), as well as between the group of 9 year-olds (p<0.01) compared with 10 and 12 year-olds, in the forward span scores, and between the groups of 6-year-olds (p<0.01), 7 year-olds (p=0.02), 8 year-olds (p=0.01), and 9 year-olds (p=0.03) compared with 12-18 year-olds, in the backward span scores.

**Figure 1:**
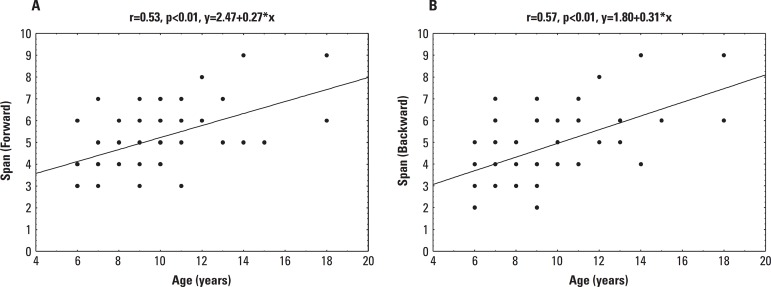
Regression lines representing Spatial Span Performances according to age, Pearson’s coefficient (r), p-values, and regression equations. [A] forward trial and [B] backward trial.

Linear regression analysis showed significant prediction for better span performances by increasing age for both forward trials and backward trials (Figure 1). Some points corresponded to more than one participant (e.g. four 6-year-old children showed the same span length on forward trial).

## Discussion

In the present study, an automated version of the spatial span task was used that is a variation on the Corsi Block-Tapping task, a manual test in which the target locations are presented as nine wooden cubes fixed to a wooden board.^8^

Our results showed no statistical difference between the sexes for span performance, although girls had a higher average than boys at ages of between 8 to 14 years on the forward span, while boys outperformed girls on the backward span. This finding corroborates previous results obtained with the Corsi Block-Tapping task in American children and young adults,^9^ as well as in Brazilian children^10^ where no statistical differences were found between boys and girls. Similarly, Postma et al.^11^ also found no statistical differences between the sexes in spatial working memory. This is also true with regard to verbal span, as concluded in the meta-analysis by Lynn and coauthors^12^ which showed no gender differences in the Wechsler digit span subtest. We believe that cognitive performance differen­ces between genders, regardless of age, are relatively small and can only be demonstrated by testing large number of subjects.

Statistical comparisons of our results as a function of years of schooling revealed no differences between forward and backward SSP. Nevertheless, we noted that scores for the backward SSP were consistently lower than for the forward SSP.

We also report age differences in performance comparing younger and older participants, showing that performance improves with increasing age.

The Rosen et al. (1997)^13^ study in undergraduate students showed that the forward and backward tasks reflect different levels of processing complexity or different types of representations. In their study however, no difference between forward and backward scores were found, implying that both tasks required a similar level of processing complexity.

Kessels and collaborators^2^ also found no differences between the forward and backward condition on the Corsi Block-Tapping Task (spatial span), but confirmed that the Digit Span (verbal span) backward was more difficult than the forward condition.

The analysis of the results as a function of educational level revealed differences in performance among participants categorized in the first years of schooling and participants in the final ranges of high school education, showing that performance improved with increased schooling, and concomitant advancement in age. This expected result had previously been demonstrated using the Corsi Block-Tapping Task in children.^10^

Forward and backward span scores were positively correlated with age, suggesting improvement in span performance with development. Luciana and Nelson^9^ suggested that memory capacity measured by the span task does not reach functional maturity by the age of 12. According to the authors, 18 years is the age at which adult levels of performance are attained on the CANTAB Spatial Span task. Indeed, the fact that the groups with 1 to 3 years of schooling committed significantly more errors than those with more than 6 years of education may reflect an influence of age. This increase in forward spatial span performance with advancing age was also found using the Corsi Block-Tapping task.^14,15^

The total number of errors is lower in reverse order because the test is stopped when the subject makes three consecutive errors. In direct order, mistakes tend to be made throughout the test and therefore the total number of trials and errors is higher. In reverse order, mistakes tend to be made successively and in the early stages of the test. Post-hoc comparisons confirmed that older children have better performance on the spatial span task. This has also been reported for other testing situations in previous studies.^2,13,15,16^

An extensive normative study was conducted by DeLuca and collaborators.^17^ These authors investigated the development of executive function over lifespan, in subjects from 8 to 64 years old, and used several subtests of the CANTAB, including the Spatial Span test. Figure 2 depicts a comparison between our results and those obtained by the cited authors.

A comparison between our results and those obtained by DeLuca and collaborators^17^ showed that the span length scores obtained by 5 out of 6 age groups in our sample were within the range of norms described in that study. As a limitation, our study sample included only one male and two females in the 15 to 19 years age group. A larger sample will provide further support for comparison.

Despite these limitations, the comparison between the present data and results from the DeLuca and collaborators^17^ study shows similarities in performance between the Brazilian children and adolescents studied here and the group of Australian participants examined by these authors, despite the socio-cultural and economic differences.

In conclusion, our results do not show statistical differences in performance on the Spatial Span Test due to gender but observed differences in performance among participants in the first years of schooling compared to those in the final ranges of high school education, show that performance improves with greater schooling, and the concomitant advancement in age. Our results also indicate a high applicability of the Spatial Span of CANTAB for the Brazilian sample. The ease of administration of the CANTAB allows its utilization independent of subjects’ culture. The study showed that the Spatial Span CANTAB test is a useful tool for assessing pediatric samples in both research and clinical settings.

## Figures and Tables

**Table 5:** Means and standard deviations for SSP by age group and gender in present study and DeLuca^17^ study.

SSP					
	Present study			DeLuca study	
	N	SPAN (SD)		N	SPAN (SD)
6-7 (years)					
Male	8	4.50 (1.19)			
Female	10	4.00 (0.94)			
8 -10 (years)					
Male	9	4.77 (1.30)		13	5.71 (0.91)
Female	19	5.21 (0.91)		16	5.56 (1.09)
11-14 (years)					
Male		5.42 (1.51)		13	6.46 (1.51)
Female	74	6.50 (2.51)		16	6.00 (1.20)
15-19 (years)					
Male		5.04 (1.50)			7.76 (1.34)
Female	12	5.50 (0.70)		1839	6.63 (1.50)
